# Exploring the shared gene signatures and mechanism among three autoimmune diseases by bulk RNA sequencing integrated with single-cell RNA sequencing analysis

**DOI:** 10.3389/fmolb.2024.1520050

**Published:** 2025-01-07

**Authors:** Xiaofang Liu, Bin Li, Yuxi Lin, Xueying Ma, Yingying Liu, Lili Ma, Xiaomeng Ma, Xia Wang, Nanjing Li, Xiaoyun Liu, Xiaohong Chen

**Affiliations:** ^1^ Department of Neurology, The Third Affiliated Hospital of Sun Yat-Sen University, Guangzhou, China; ^2^ Department of Infectious Diseases, The Third Affiliated Hospital of Sun Yat-sen University, Guangzhou, China; ^3^ Department of Neurology, The Sixth People’s Hospital of Huizhou City, Huizhou, China; ^4^ Department of General Medicine, The Third Affiliated Hospital of Sun Yat-sen University, Guangzhou, China

**Keywords:** multiple sclerosis (MS), systemic lupus erythematosus (SLE), rheumatoid arthritis (RA), WGCNA, bioinformatics, DEGs, shared genes

## Abstract

**Background:**

Emerging evidence underscores the comorbidity mechanisms among autoimmune diseases (AIDs), with innovative technologies such as single-cell RNA sequencing (scRNA-seq) significantly advancing the explorations in this field. This study aimed to investigate the shared genes among three AIDs—Multiple Sclerosis (MS), Systemic Lupus Erythematosus (SLE), and Rheumatoid Arthritis (RA) using bioinformatics databases, and to identify potential biomarkers for early diagnosis.

**Methods:**

We retrieved transcriptomic data of MS, SLE, and RA patients from public databases. Weighted Gene Co-Expression Network Analysis (WGCNA) was employed to construct gene co-expression networks and identify disease-associated modules. Functional enrichment analyses and Protein-Protein Interaction (PPI) network was constructed. We used machine learning algorithms to select candidate biomarkers and evaluate their diagnostic value. The Cibersort algorithm was and scRNA-seq analysis was performed to identify key gene expression patterns and assess the infiltration of immune cells in MS patients. Finally, the biomarkers’ expression was validated in human and mice experiments.

**Results:**

Several shared genes among MS, SLE, and RA were identified, which play crucial roles in immune responses and inflammation regulation. PPI network analysis highlighted key hub genes, some of which were selected as candidate biomarkers through machine learning algorithms. Receiver Operating Characteristic (ROC) curve analysis indicated that some genes had high diagnostic value (Area Under the Curve, AUC >0.7). Immune cell infiltration pattern analysis showed significant differences in the expression of various immune cells in MS patients. scRNA-seq analysis revealed clusters of genes that were significantly upregulated in the single cells of cerebrospinal fluid in MS patients. The expression of shared genes was validated in the EAE mose model. Validation using clinical samples confirmed the expression of potential diagnostic biomarkers.

**Conclusion:**

This study identified shared genes among MS, SLE, and RA and proposed potential early diagnostic biomarkers. These genes are pivotal in regulating immune responses, providing new targets and theoretical basis for the early diagnosis and treatment of autoimmune diseases.

## Graphical Abstract

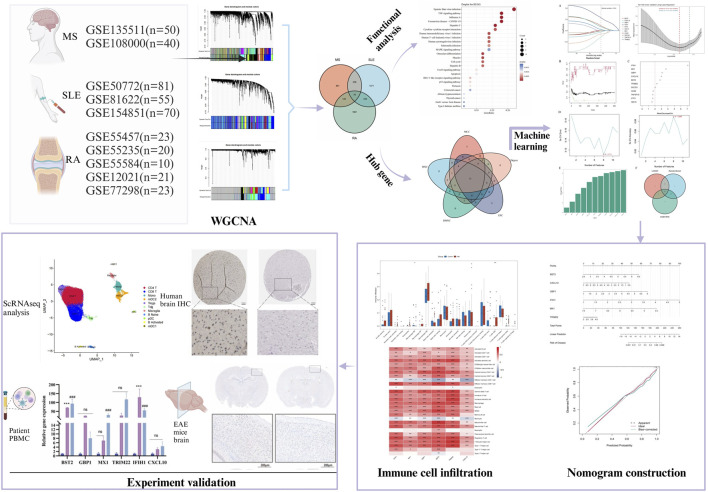

## Introduction

Multiple sclerosis (MS) is an immune-mediated inflammatory demyelinating disease of the central nervous system (CNS), predominantly affecting young and middle-aged women. Despite advancements in understanding its pathogenesis, the precise mechanisms remain unclear (Reich et al., 2018). Furthermore, the unpredictable disease course and the potential for severe complications underscore the urgency in developing effective strategies to diagnose and treat MS. Comorbidity is an area of increasing interest in MS as evidence emerges that comorbidity is associated with diagnostic delays, disability progression, health-related quality of life, and progression of lesion burden on magnetic resonance imaging (MRI) (Lo et al., 2021).

Autoimmune diseases (AIDs) are a series of conditions caused by defects of the human immune system characterized by an inability to recognize auto-antigens and subsequent pathological responses. MS, systemic lupus erythematosus (SLE), rheumatoid arthritis (RA), psoriasis, and type I diabetes (T1DM) are common AIDs. These diseases affect 3%–8% of the general population, with women making up 78%–85% of those affected. The potential for shared pathogenic mechanisms among AIDs is suggested by the significant presence of non-organ-specific autoantibodies in conditions like Klinefelter syndrome (47,XXY), which is closely associated with rheumatic diseases (Rovenský, 2006; Seminog et al., 2015; Panimolle et al., 2021). Compared to the 46,XY control group, there is evidence of increased organ-specific autoimmune conditions in individuals with severe and typical X chromosome aneuploidies. This commonality in pathogenesis is further supported by the significantly increased risk for various autoimmune diseases in KS patients, including 7-Addison’s disease, T1DM, MS, acquired hypothyroidism, RA, Sjögren’s syndrome, and SLE (Panimolle et al., 2018). In the pathogenesis of autoimmune diseases, certain genetic predispositions and environmental triggers are common, exhibiting shared immune dysregulation disorders. Most AIDs is systemic, while some primarily affect a single organ or structure. Rarely, a few AIDs coexist in one person, which can suggest similar pathogenetic mechanisms (Belniak et al., 2007). Complex interactions between genetic, infectious and/or environmental factors probably contribute to the presence of these diseases. Although the detailed pathogenesis varies in specific AIDs, multiple cellular and molecular mechanisms are considered to be shared among them (Liu et al., 2024). Understanding these shared mechanisms can facilitate the discovery of novel diagnostic biomarkers and therapeutic targets, potentially leading to more effective and personalized treatment regimens for patients.

The most frequently studied comorbidities were RA and SLE, as well as psychiatric conditions, and so on (Marrie et al., 2015). MS-associated immune cells include CD4^+^ T lymphocytes, CD8^+^ T lymphocytes, B cells, innate lymphoid cells (ILCs), NK cells, monocytes, macrophages, dendritic cells and so on (Attfield et al., 2022). SLE is an autoimmune disease that causes chronic inflammation and is associated with the production of autoantibodies, with a prevalence rate of 0.02%–0.15% worldwide (Kuo et al., 2015; Rees et al., 2016). RA is a systemic autoimmune disease characterized by arthropathy (Weyand and Goronzy, 2017). As immune-mediated diseases, the pathogenesis of RA and SLE is closely related to different immune cells (Zampeli et al., 2015). These cells secrete pro-inflammatory factors and proteases that could destroy cartilage and bone (Fang et al., 2020). Chronic inflammation in the articular joints leads to joint and bone destruction in RA, whereas uncontrolled production of autoantibodies against nuclear antigens leads to systemic inflammation in SLE (Fang et al., 2021). A cross-sectional analysis of Australian MS Longitudinal Study (AMSLS) participants (n = 902) showed that SLE, individual comorbidities, were most strongly associated with overall health-related quality of life (HRQoL), and SLE, RA and hyperthyroidism with physical HRQoL. Comorbidities potentially make important contributions to HRQoL in MS (Lo et al., 2021). Many case reports have shown a coexistence of MS with SLE (Hietaharju et al., 2001; Fanouriakis et al., 2014; Jácome Sánchez et al., 2018). A study found that the RA inflammation subtype, and the MS “inflammation and EGF” subtype share similarities, which display a consistent pattern of inflammation driven by the activation of the JAK-STAT pathway (Cheng et al., 2024). MS can coexist with RA and may potentially impact treatment medications (Hojjati et al., 2016; Brummer et al., 2021). Some studies have also found links and patterns of comorbidities among various types of AIDs, including lupus (SLE and RA), MS, and T1DM (Wen and Yu, 2023). Furthermore, identifying new diagnostic and detection targets, as well as pathophysiological biomarkers for dual autoimmune diagnoses, may help to uncover shared key genes and biological pathways between dual AIDs. The shared genetic loci associated with RA and SLE are *tumor necrosis factor receptor-associated factor (TRAF1), tumor necrosis factor receptor superfamily member 5 (CD40/TNFRSF5), TNF-α-induced protein 3 (TNFAIP3), interferon regulatory factor 5 (IRF5), B lymphoid tyrosine kinase (BLK)*. The shared genetic loci associated with RA and SLE are *interleukin 12A (IL-12A), ribosomal protein L19 pseudogene 8 (RPL19P8), CD40/TNFRSF5, IRF8*. The shared genetic loci associated with RA and MS include *kinesin family member 5A protein (KIF5A), DNAX accessory molecule-1(DNAM-1)/CD226* (Ahmad and Ahsan, 2022).

The coexistence mechanisms of SLE, RA and MS can pose challenges to the diagnosis, therapies, and prognosis in clinical practice. However, previous efforts primarily focused on identifying diagnostic biomarkers of MS, SLE, and RA, respectively, and the exact relationship between MS and SLE, RA has not been fully established. Therefore, investigating the comorbidity mechanisms of MS, SLE, and RA is of significant clinical importance for early recognition and intervention. The development of current transcriptome and single-cell sequencing technologies provides multidimensional clues for us to study the connections between these diseases. Using integrated bioinformatics approaches and machine learning algorithms, we sought to find potential early diagnostic biomarkers for MS incidence in patients with SLE and RA and clarify the immunological mechanisms. We also illustrated the hub gene expression profiles in single cells from MS patients. Our findings highlight groups of and individual comorbidities that could provide the largest benefits for MS with RA or SLE if they were targeted for prevention, early detection, and optimal treatment. These genes may serve as potential therapeutic targets and biomarkers, offering significant promise for clinical practice.

## Materials and methods

### Microarray data

**FIGURE 1:**
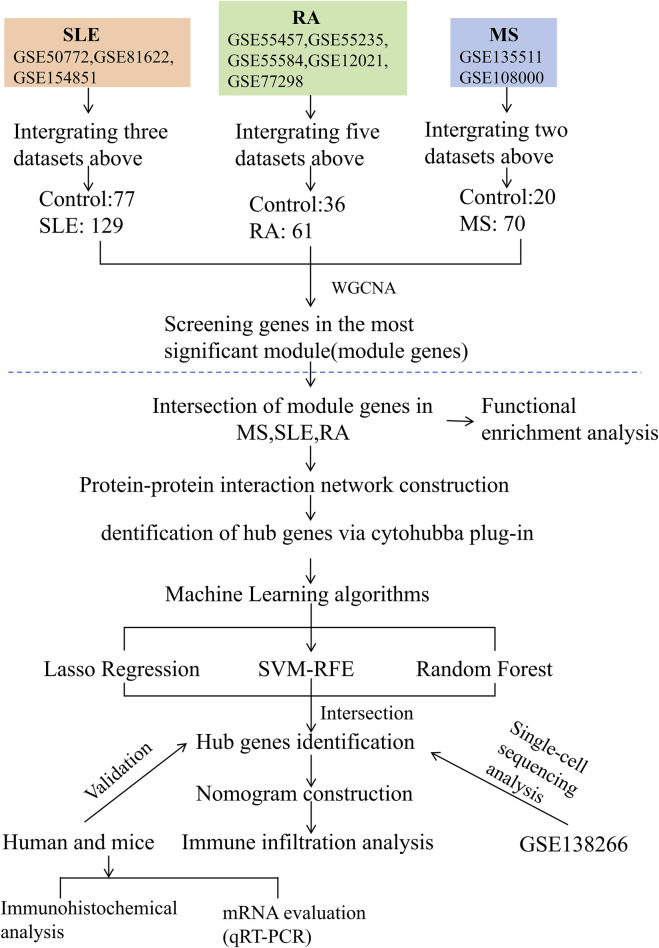
Flowchart illustrating the study design.

The study design is depicted as a flowchart in Figure 1. Ten publicly available microarray datasets (GSE135511, GSE108000, GSE50772, GSE81622, GSE154851, GSE55457, GSE55235, GSE55584, GSE12021 and GSE77298) representing transcriptome profiles were retrieved from the NCBI Gene Expression Omnibus database (GEO) (Barrett et al., 2013) (https://www.ncbi.nlm.nih.gov/geo/). Detailed information about the datasets is given in Table 1. Multiple GEO data sets were combined for analysis. The ComBat function, which is based on the classical Bayesian framework, was applied to remove batch effects among different datasets via the sva package (Johnson et al., 2007).

**TABLE 1:** Detailed information on transcriptome datasets used in the study.

ID	GEO accession No	Disease	Control case	Sample size	Sourcetypes	Platform	Experiment type
1	GSE135511	MS	10	40	brain	GPL6883	Expression profiling by array
2	GSE108000	MS	10	30	brain	GPL13497	Expression profiling by array
3	GSE50772	SLE	20	61	Peripheral Blood	GSE50772	Expression profiling by array
4	GSE81622	SLE	25	30	Peripheral Blood	GPL10558	Expression profiling by array
5	GSE154851	SLE	32	38	Peripheral Blood	GPL16699	Expression profiling by array
6	GSE55457	RA	10	13	synovium	GPL96	Expression profiling by array
7	GSE55235	RA	10	10	synovium	GPL96	Expression profiling by array
8	GSE55584	RA	0	10	synovium	GPL96	Expression profiling by array
9	GSE12021	RA	9	12	synovium	GPL96、GPL97	Expression profiling by array
10	GSE77298	RA	7	16	synovium	GPL570	Expression profiling by array

MS, multiple sclerosis; SLE, system lupus erythematosus; RA, rheumatoid arthritis.

### Significant module identification via weighted gene coexpression network analysis (WGCNA)

WGCNA has been widely applied to construct a gene coexpression network (Langfelder and Horvath, 2008). Herein, we adopted WGCNA to identify significant module genes highly correlated with SLE. First, every gene’s median absolute deviation (MAD) was calculated, and 50% of genes with the smallest MAD were eliminated. Subsequently, a scale-free coexpression network was constructed by filtering the differentially expressed genes (DEGs) expression matrix using the goodSamplesGenes function in WGCNA. Next, the adjacency was calculated based on the soft thresholding power β, derived from coexpression similarity, using the pick-Soft-Threshold function. Then, the adjacency was converted into a topological overlap matrix (TOM), followed by the calculation of the gene ratio and corresponding dissimilarity (1-TOM). Hierarchical clustering and dynamic tree-cutting were then used to identify modules. To classify genes with similar characteristics, TOM-based dissimilarity measures with a minimum size (gene group) of 50 were used for the gene dendrogram, and an average linkage hierarchical clustering was initiated. Finally, we chose a cut-off for the module dendrogram, and some modules were merged based on the dissimilarity of estimated module eigengenes. The eigengene network was visualized with the “TOMplot” function of WGCNA with heatmap.

### Enrichment analyses of overlapping shared genes

During Gene Ontology (GO) analysis, biological process (BP), cellular component (CC) and molecular function (MF) are identified (The Gene Ontology Consortium, 2019). The R package “ClusterProfiler” was used to conduct functional enrichment analysis, and the top 10 GO terms were visualized using the R package “ggplot2″ in each category based on the screening criteria: false discovery rate (FDR) < 0.05 and adjusted *p*-value < 0.05 (Yu et al., 2012). Kyoto Encyclopedia of Genes and Genomes (KEGG) analysis and Reactome functional enrichment analyses were conducted and visualized.

### Protein-protein interaction (PPI) network construction and candidate hub genes selection

Shared genes were mapped to the PPI network to further explore their potential interplay. The PPI network was constructed using the Search Tool for Retrieval of Interacting Genes (String) database (Szklarczyk et al., 2021) (version 11.5; www.string-db.org), with a minimum required interaction score of 0.400. The genes that did not interact with each other were hidden. Cytoscape software (Otasek et al., 2019) was used to visualize the PPI network. To identify hub genes, the Cytoscape plug-in CytoHubba was used for topological analysis using the five different algorithms:Degree, Maximal clique Centrality (MCC), Maximum Neighborhood Component (MNC), Density of Maximum Neighborhood Component (DMNC), and Edge percolated component (EPC) (Niu et al., 2020). Importantly, nodes can be measured based on their network features to determine their importance in biological networks and identify central elements of biological networks. Finally, the top 30 DEGs obtained from the intersection of the three algorithms were visualized.

### Machine learning algorithms

To further identify candidate biomarkers, three machine learning algorithms were applied: least absolute shrinkage and selection operator (LASSO) regression, random forest, and support vector machine-recursive feature elimination (SVM-RFE). For the diagnostic value assessment in this study, the intersection of shared genes filtered by all 3 machine learning algorithms was chosen.

### Nomogram construction

The expression of each DEG between MS/SLE and the control group in the dataset was compared using the Student’s t-test. To evaluate the predictive value of each candidate biomarker, we generated ROC curves and calculated the area under the curve (AUC) and 95% confidence interval (CI). After that, the nomogram was generated using the R package “rms”. Each gene’s relative expression level corresponds to a score based on the nomogram. The summation of each score was referred to as the total score, which could be used to predict the incidence of SLE with AS. Meanwhile, we constructed the ROC curve of the nomogram. The optimal AUC for predicting the risk of SLE with AS was >0.7.

### Immune cell infiltration pattern analysis

The algorithm “Cibersort” can transform the normalized gene expression matrix into the infiltrating immune cell composition. The R package “Cibersort” has previously been used to quantify the proportions of 22 kinds of immune cells between MS and control (Newman et al., 2015). The proportion of each immune cell in each sample was visualized from the barplot. The comparison of expression of the difference regarding each immune cell between the two groups was displayed in a boxplot. A heatmap displaying the correlation of different immune cells in MS pathogenesis was constructed using the R package “corrplot” (Wu et al., 2024).

### Single-cell RNA-sequencing (scRNA-seq) analysis

The scRNA-seq data of GC was accessed from the GSE138266 dataset, which is established in the GEO database corresponding to a published article “Integrated single cell analysis of blood and cerebrospinal fluid leukocytes in multiple sclerosis”. The raw gene expression matrix was imported and processed using the Seurat package (version 4.4.0). First, we filtered out cells expressing fewer than 200 genes and genes expressed in fewer than 3 cells. The percentage of mitochondrial genes was evaluated using the PercentageFeatureSet function. Cells with >200 and <2,500 expressed genes were kept and those with below 5% mitochondrial content were removed. Then an expression matrix comprising 33,879 cells and 19,650 genes was generated for further analysis. Next, the FindVariableFeatures function was used to discover hypervariable genes. The data were normalized using the ScaleData function, and principal component analysis (PCA) was then performed with 30 PCs selected. The RunHarmony function was applied for data integration to remove the batch effect between samples. Subsequently uniform manifold approximation and projection (UMAP) analysis was used to reduce the dimensions. Cell types within the obtained clusters were annotated by the cell marker genes from previous studies (Schafflick et al., 2020) and the CellMarker database (http://117.50.127.228/CellMarker/).

### Animals and EAE induction

Six-to eight-week-old female C57BL/6 mice, weighing 16–20 g, received subcutaneous injections of 200 μg Myelin Oligodendrocyte Glycoprotein 35–55 (MOG35-55) emulsion at both the upper and lower back (100 μL per injection site) to induce EAE. The emulsion, prepared under sterile conditions, included MOG35-55 (purity > 95%, Guidechem, Shanghai, China) in 200 μL of complete Freund’s adjuvant (CFA, Sigma F5881, United States), containing *Mycobacterium tuberculosis* (5 mg/mL; strain H37Ra, BD 231141, United States) and emulsified with phosphate-buffered saline (PBS). All animals were administered 250 ng pertussis toxin (List Biological Laboratories, PTX181, United States) intraperitoneally on the day of immunization and on the second day post-initial injection. Daily weight measurements and clinical symptom monitoring for EAE were conducted, following this scale: grade 5, death; grade 4.5, near death, moribund; grade 4, complete paralysis of two limbs; grade 3, complete paralysis of a single limb; grade 2.5, partial limb paralysis and ataxia; grade 2, dysfunctional gait with limp tail and ataxia; and grade 1, dysfunctional gait with tail tonicity or limp tail. Intermediate clinical signs were scored by adding a value of 0.5. All experimental protocols were approved by the Animal Care and Use Committee of South China Agricultural University (Approval ID: 2022D157) in adherence with the Guide for the Care and Use of Laboratory Animals and in compliance with the ARRIVE guidelines.

### Clinical samples collection

The selection of SLE patients is based on The 2019 SLE EULAR/ACR classification standards (Assan et al., 2021) and RA patients is based on The 1987 ACR guidelines (Arnett et al., 1988). The study was approved by the Ethics Committee of the Third Affiliated Hospital of Sun Yat-sen University (Ethical number: II 2024-184–01) and was performed in accordance with the Declaration of Helsinki. Written informed consent was obtained from all participants. The selection criteria for the screening samples were defined as follows: 1) Samples were exclusively derived from a single hospital; 2) Each cohort comprised no fewer than 10 individuals; 3) Participants were newly identified with RA or SLE, admitted for inpatient care with a confirmed diagnosis, exhibiting pronounced clinical signs, and presenting at the earliest phase of their illness without a history of other concurrent diseases; 4) Patient specimens were obtained promptly upon definitive diagnosis. Prior to sample collection for this research, no study subjects had received any form of medical treatment. The control group were selected from a pool of healthy volunteers at the Third Affiliated Hospital of Sun Yat-sen University.

PBMCs were isolated by density-gradient centrifugation with the Lymphoprep Premium kit (STEMCELL Technologies) according to the manufacturer’s instructions. The cells were resuspended in 10% fetal bovine serum containing RPMI 1640.

### RNA isolation and quantitative real-time polymerase chain reaction (qRT- PCR)

TRIzol reagent (Vazyme, Nanjing, China) was used to extract RNA from PBMC samples, and cDNA was synthesized using HiScript III RT SuperMix for qPCR (Vazyme, Nanjing, China). Then RT-PCR was performed to quantitative the expression level of GLIPR1 and MAMLD1 in the two diseases. Primer sequences of the two genes were described in Supplementary Table S1.

### Verifcation and localization of predictive genes

The immunohistochemical (IHC) data were downloaded from the Human Protein Atlas (HPA) database (https://www.proteinatlas.org/) to determine the localization characteristics of key genes in human cortex. We further verifed the expression profle of five key genes in the EAE mice brain by immunohistochemical.

### Statistical analysis

R software version 4.2.1, GraphPad Prism Version 9.4.0 (GraphPad Software, San Diego, CA, United States) and SPSS Version 26.0 (IBM Corporation, Armonk, NY, United States) were used to perform statistical analyses. Using the Student’s t-test, continuous variables were compared between the two groups. A *p*-value < 0.05 was statistically significant.

## Results

### WGCNA analysis identified significant module genes in MS, SLE and RA

**FIGURE 2:**
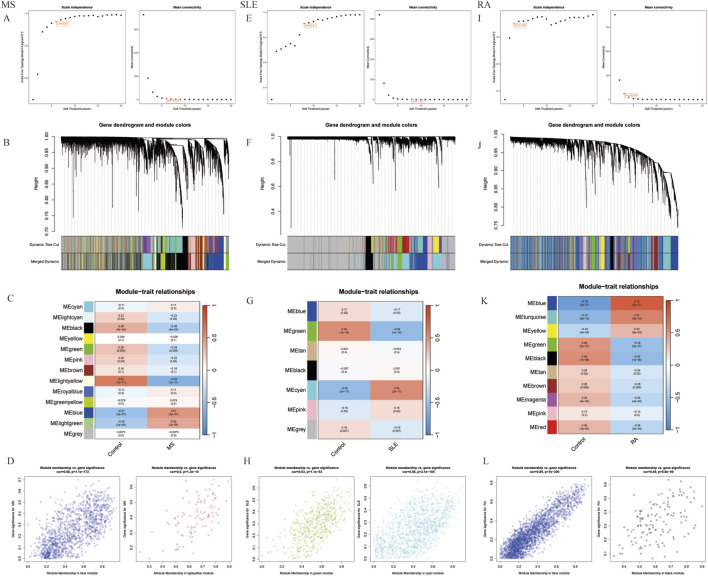
Selection of gene modules associated with MS with WGCNA. **(A)** Soft thresholding power (β) selection via scale independence and average connectivity. **(B)** Gene clusters, or modules, associated with MS are shown in different colors under the cluster dendrogram. **(C)** The heatmap depicting the correlation between gene modules and MS. The top digit represents the correlation coefficient, and the bottom digit shows the P-value. **(D)** The scatter plot showing the correlation between module membership and gene significance in MS regarding the most positively (blue) and negatively (light yellow) correlated modules. **(E)** Identifying the soft-threshold power in SLE. **(F)** Cluster dendrogram displaying highly connected genes in key modules associated with SLE. **(G)** Interconnections among modules and traits in SLE. Correlation coefficients and P values are incorporated in each cell. **(H)** The scatter plot showing the correlation between module membership and gene significance in SLE regarding the most positively (green) and negatively (cyan) correlated modules. **(I)** Estimation of the soft-threshold power for RA. **(J)** Dendrogram clustering of RA modules featuring genes with strong connectivity. **(K)** Associations between modules and traits in RA. **(L)** The scatter plot showing the correlation between module membership and gene significance in RA regarding the most positively (Blue) and negatively (black) correlated modules. WGCNA, weighted gene co-expression network analysis.

Using WGCNA, significant module genes of MS were identified. The Gray module was regarded as a junk module, meaning it failed to cluster genes. The most acceptable soft thresholding power β = 6 was chosen based on scale independence and average connectivity (Figure 2A). Following module merging, 13 gene coexpression modules related to MS were obtained, shown in different colors (Figures 2B,C). The colors depict the relationship between the modules and MS, with blue having the strongest positive (1,267 genes; correlation coefficient (CC = 0.51; P = 3 × 10^−7^) and lightyellow the strongest negative correlation (95 genes; CC = −0.62; P = 7 × 10^−11^) with MS. Furthermore, a significant correlation between blue (r = 0.68) and lightyellow (r = 0.6) module membership and gene significance for MS was observed (Figure 2D). Therefore, the 1,362 genes in the blue and lightyellow modules most associated with MS were recognized as crucial for further experiments. Similarly, WGCNA was applied to the SLE and RA groups. WGCNA identified 1739 SLE module genes with the strongest positive green module and the strongest negative cyan module (Figures 2E–H). Similarly, WGCNA-identified 2,422 module genes associated with RA (Figures 2I–L).

### Functional enrichment analysis of shared genes in MS, SLE and RA

**FIGURE 3:**
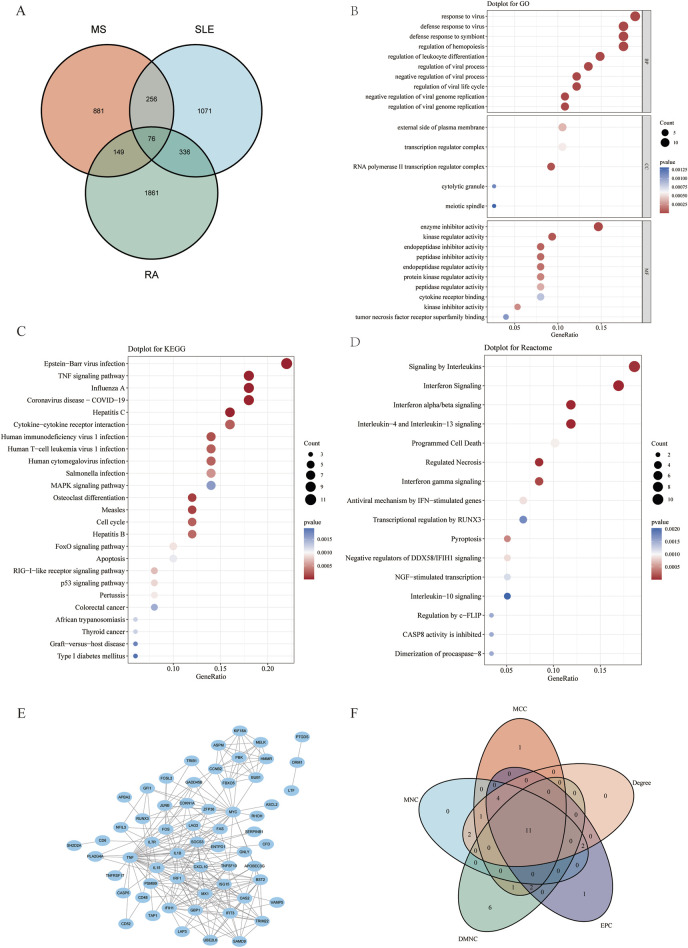
Functional enrichment analysis of shared genes associated with MS,SLE and RA and the node gene selection from PPI network. **(A)** The Venn plot displays that the intersection of significant module genes in MS, significant module genes in SLE and significant module genes in RA yielded 76 DEGs. **(B–D)** GO analysis (BP, CC, MF), KEGG pathway analysis and Reactome analysis of shared genes. The X-axis represents the gene ratio, Y-axis refers to different ontologies, the circle size represents the gene number, and the color indicates the significance. **(E)** The whole PPI network of 76 DEGs was visualized via STRING. **(F)** 11 DEGs were selected for further analysis based on the intersection of genes from five algorithms. GO, gene ontology; KEGG, Kyoto Encyclopedia of Genes and Genomes. PPI, Protein-protein interaction.

To investigate the co-pathogenesis of MS, SLE and RA, 76 shared genes (Figure 3A) were selected from the genes identified by WGCNA. Functional enrichment analyses of the 76 overlapping shared genes were performed with GO, KEGG and Reactome analysis. As shown in Figure 3B, the process includes: 1) biological process, response to virus, defense response to virus, regulation of leukocyte differentiation; 2) cellular component, external side of plasma membrane, transcription regulator complex, RNA polymerase II transcription regulator complex; and 3) molecular function, enzyme inhibitor activity, cytokine receptor binding, and tumor necrosis factor receptor superfamily binding. The KEGG pathway enrichment analysis revealed that these genes were primarily enriched in Epstein-Barr virus infection, TNF signaling pathway, and cytokine-cytokine receptor interaction (Figure 3C). Furthermore, the Reactome enrichment analysis revealed that these core genes were significantly abundant in Epstein-Barr virus associated Interferon signaling (Figure 3D). These results suggest that the overlapping DEGs participate in response to virus, Interferon signaling, inflammation and immunological response in MS, SLE and RA.

### PPI network construction and hub gene selection of intersection of significant module genes of MS, SLE, and RA

Based on the 76 shared genes, a PPI network was preliminarily constructed to select hub genes for MS. After eliminating DEGs with poor interaction (n = 3), 73 genes were retained (Figure 3E). Moreover, to identify the top 20 intersected genes, we used five different algorithms (Degree, MCC, MNC, DMNC, and EPC) in the Cytoscape plug-in CytoHubba (Figure 3F). Finally, 11 genes were selected using Venn plots for further machine learning analysis.

### Selection of candidate diagnostic biomarkers of MS progression using machine learning

**FIGURE 4:**
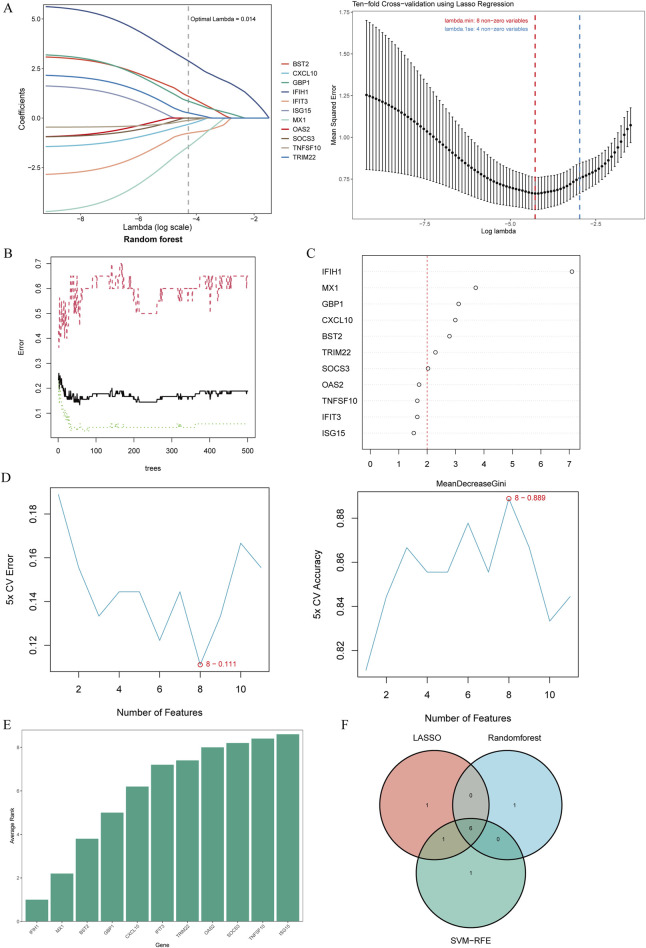
Candidate biomarker identification via machine learning algorithms. **(A)** Based on the Lasso regression algorithm, 8 genes were identified as the biomarkers with the lowest binominal deviation. **(B)** The diagnostic error relating to MS,SLE and RA was visualized from the random forest. **(C)** The column showing 11 DEGs ranked based on the importance score calculated from the random forest. **(D)** 11 genes were selected based on SVM-RFE with the lowest error and highest accuracy. **(E)** DEGs were ordered based on the average rank from SVM-RFE. The lower rank indicates positions of higher importance. **(F)** The intersection of 3 machine learning algorithms was obtained with a Venn diagram tool, yielding 6 DEGs selected as the candidate biomarkers. LASSO, least absolute shrinkage and selection operator; SVM-RFE, support vector machine recursive feature elimination; AMI, acute myocardial infarction.

To select shared genes for diagnostic evaluation, three different machine learning algorithms were applied. LASSO regression analysis revealed 8 genes with the lowest binomial deviance among the top 30 node shared genes (Figure 4A). After ranking the shared genes according to the gene importance score, the random forest method was applied, recognizing 7 potential candidates (Figures 4B,C). Furthermore, the SVM-RFE approach uncovered 8 genes with the lowest error and highest accuracy after 100 folds for diagnosing MS progression in SLE and RA (Figures 4D,E). Finally, the DEGs detected by each method (LASSO, n = 8; random forest, n = 7; and SVM-RFE, n = 7) were intersected and 6 genes were visualized as a Venn diagram: *Bone marrow stromal antigen 2 (BST2), Guanylate binding protein 1 (GBP1), MX Dynamin Like GTPase 1 (MX1), Interferon-induced with helicase C domain 1 (IFIH1), Tripartite Motif Containing 22 (TRIM22), C-X-C Motif Chemokine Ligand 10 (CXCL10)* (Figure 4F).

### Assessment and validation of biomarker diagnostic value and nomogram construction

**FIGURE 5:**
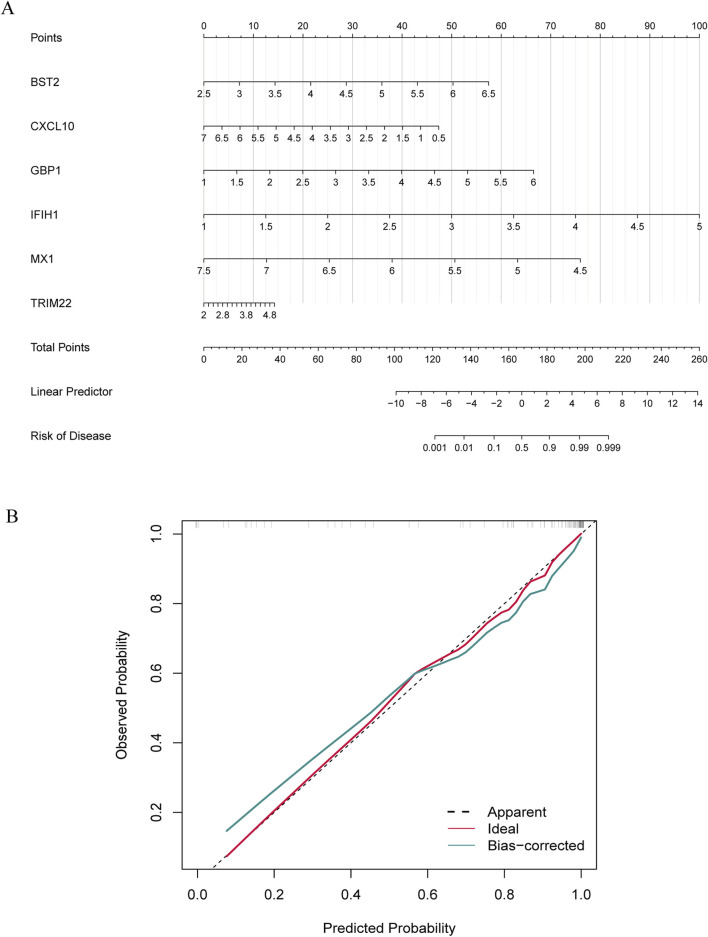
Nomogram construction and diagnostic performance validation. **(A)** The nomogram was established based on the 6 selected candidate biomarkers. Each gene corresponds to a score. The total score of the 6 DEGs is used to predict the risk of MS in a population with MS and RA. **(B)** The calibration curve of nomogram model prediction in MS with SLE and RA. The red solid line is marked as “Ideal”, which represents the standard curve, and is on behalf of the perfect prediction of the ideal model. The dotted line is marked as “Apparent”, which indicates the uncalibrated prediction curve, while the blue solid line is marked as “Bias-corrected” and represents the calibrated prediction curve.

To further explore the potential of these core shared DEGs as clinical biomarkers in the disease, the ROC curves of 6 candidate biomarkers were generated, and AUCs were calculated for the candidates to assess their diagnostic value: *BST2* (AUC, 0.779; 95% CI, 0.678–0.881); *GBP1* (AUC, 0.809; 95% CI, 0.712–0.907); *MX1* (AUC, 0.519; 95% CI, 0.38–0.658); *TRIM22* (AUC, 0.752; 95% CI, 0.636–0.868); *IFIH1* (AUC, 0.867; 95% CI; 0.783–0.951); *CXCL10* (AUC, 0.604; 95% CI, 0.436–0.771) (Supplementary Figure S1A). The candidate expression was also evaluated in the GSE123496 validation dataset. Moreover, the AUCs for these 4 genes were <0.7, while they were above this value for the remaining 2 (Supplementary Figure S1B). These 6 genes were ultimately selected to construct a nomogram, after multiple rounds of selection (Figure 5A). The relative expression level of each gene corresponded to a score in the nomogram. Finally, the overall score was used to forecast the incidence of MS progression in patients with SLE and RA. The calibration curves uncovered that the predicted probability of the constructed nomogram diagnostic model was almost identical to that of the ideal model (Figure 5B).

### Immune infiltration analysis

**FIGURE 6:**
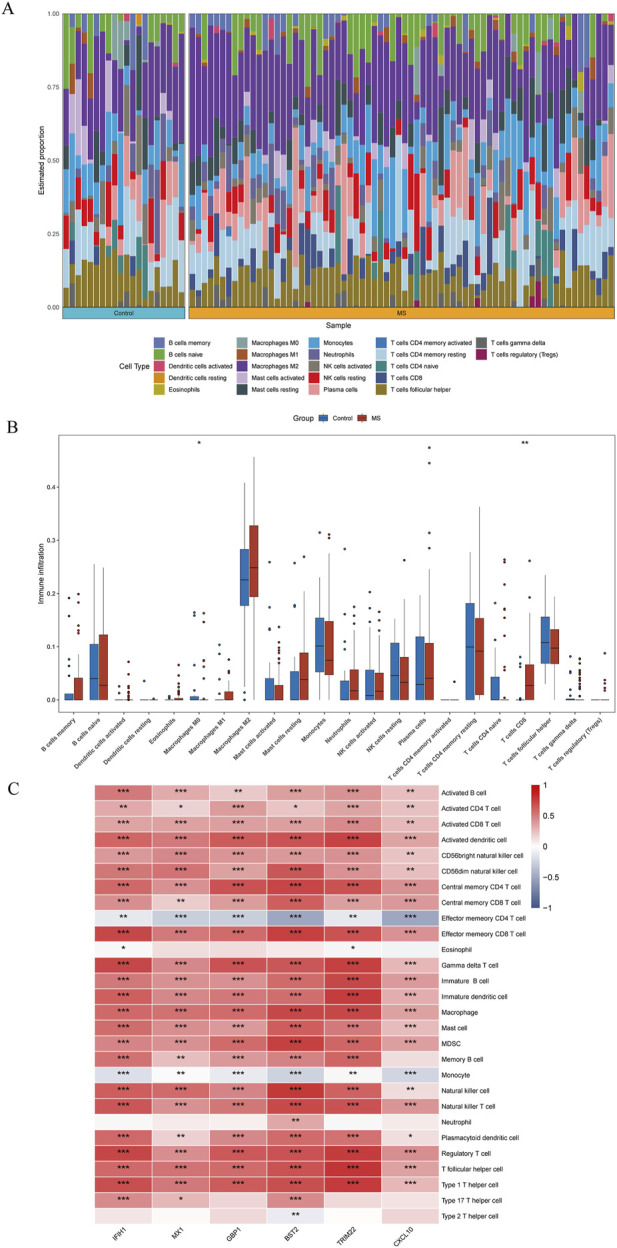
Quantification of immune cell infiltration in MS. **(A)** The relative proportion of 22 types of immune infiltrating cells in MS samples is shown as a barplot. **(B)** The boxplot compares the expression of immune cells between MS and controls. **(C)** Correlation analysis of immune cell infiltrations with six shared genes. *p < 0.05; **p < 0.01; ***p < 0.001; ****p < 0.0001.

Immune infiltration is a significant pathophysiological characteristic and is associated with MS progression. To explore the relationship between candidate biomarkers and MS, we conducted the immune infiltration analysis. A barplot was generated to visualize the proportions of immune cells in each sample. The barplot (Figure 6A) showed that the proportion of memory B cells, M1 Macrophages, resting Mast cells, and CD8^+^ T cells were higher in MS, while activated Mast cells, Monocytes, resting NK cells, memory resting CD4^+^ T cells, naive CD4^+^ T cells, and M0 Macrophages were lower in MS compared with control (Figure 6B). Moreover, we found a positive correlation between the six shared genes and immune cell infiltrations (Figure 6C).

### The expression level of key signatures based on scRNA-seq analysis

**FIGURE 7:**
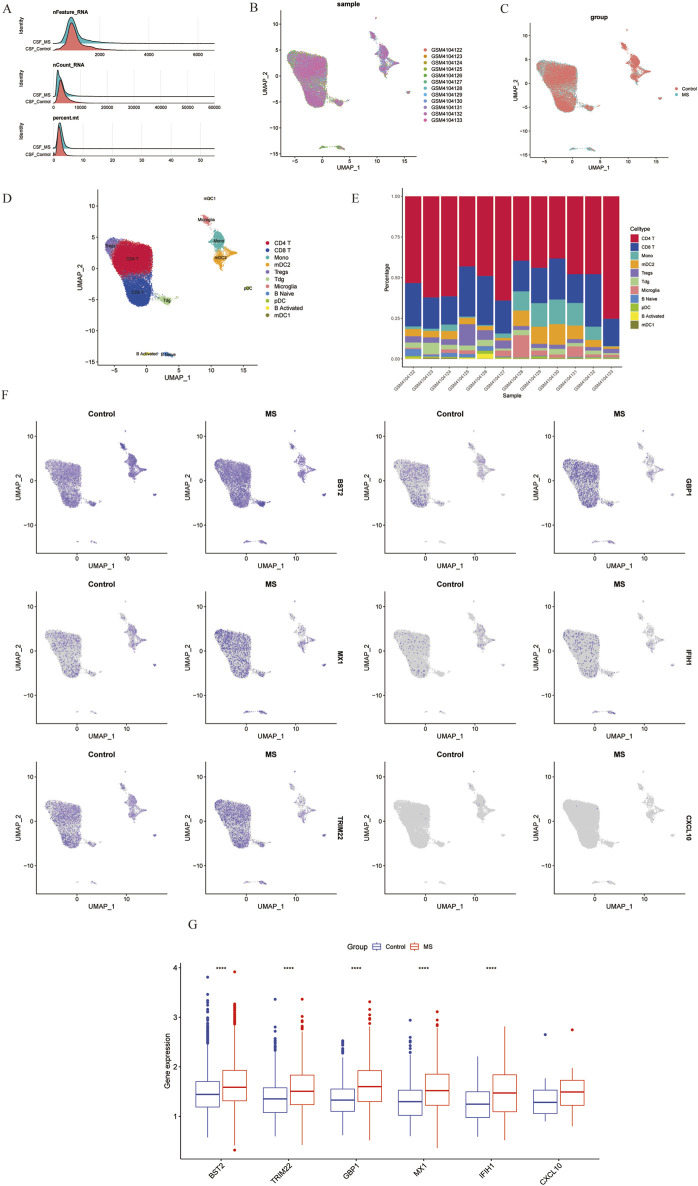
Overview of single-cell atlases of normal and MS samples. **(A)** Quality control of scRNA-seq data of samples of HCC cells. **(B)** UMAP visualization of clustering plot of 10 samples. **(C)** UMAP visualization of clustering plot comparing control and MS tissues. **(D)** UMAP visualization of plot depicting clustering of single-cell samples into 11 clusters. **(E)** Proportional representation of different cell types in 10 sampes. **(F)** UMAP plot highlighting the expression patterns of marker genes for the 11 cell types. **(G)** Boxplots displaying expression of marker genes for the 8 cell types across cells. UMAP, Uniform Manifold Approximation and Projection.

In this study, we analyzed a total of 10 samples from the GSE138266 dataset, including CSF and PBMC from 5 patients and 5 controls. The number of genes (nFeature), the sequence count per cell (nCount), and percentage of mitochondrial genes (percent.mt) were displayed in Vlnplots (Figure 7A). Figure 7B illustrates the single-cell transcriptome atlas across different samples, while Figure 7C displays the single-cell transcriptome atlas for control and MS samples. Cell annotation using Single R package revealed 11 cell types (Figure 7D): Tdg, Tregs, CD4^+^ T, CD8^+^ T, B Naïve, B Activated, pDC, mDC1, mDC2, Mono, and Microglial. Figure 7F shows the expression patterns of shared genes for each cell subtypes in control and MS, including *BST2, GBP1, MX1, IFIH1, TRIM22, and CXCL10.* Figure 7G shows the comparison of the contents of the above six genes in the control group and the MS group, and there was no statistical difference in the content of the *CXCL10* gene. Figure 7E represents their proportions across different samples, with CD4^+^ T cells and CD8^+^ T cells exhibiting higher proportions, while Tdg, Tregs, B Naïve, B Activated, pDC, mDC1, mDC2, and Microglial constituted a relatively smaller proportion.

### Expression of six shared genes in human and mice

**FIGURE 8:**
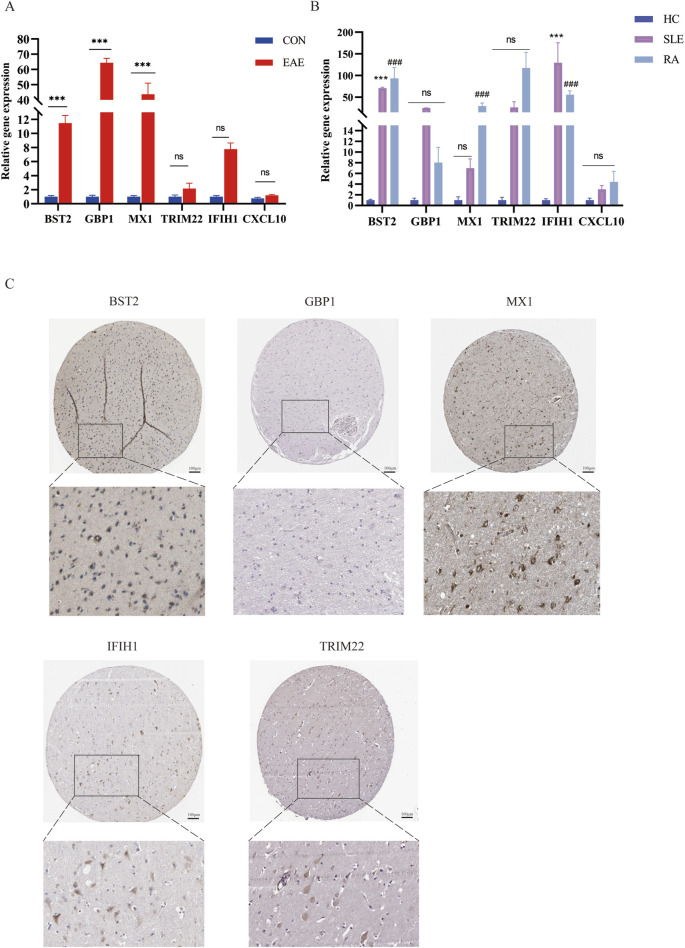
The mRNA expression of six shared genes in human and mice. **(A)** qRT-PCR showing increased mRNA levels of *BST2, GBP1, MX1, IFIH1, TRIM22, and CXCL10* in the spinal cord of EAE mice. **(B)** The expression of six hub genes in SLE and RA patients. qRT-PCR showing increased mRNA levels of *BST2, GBP1, MX1, IFIH1, TRIM22 and CXCL10* in PBMC of SLE and RA patients. **(C)** IHC results in the expression of the five key genes for normal people cortex in the HPA database. HPA, Human Protein Atlas.

We used the MOG35-55 induced EAE mouse model, the most common MS mouse model, to isolate the lumbar spinal segment of mice, and assessed the expression of the above six genes by qRT-PCR. The results showed that *BST2, GBP1,* and *MX1* mRNA were increased in the spinal cord of EAE mice compared with the control group with statistical significance. As for *IFIH1, TRIM22,* and *CXCL10*, although their mRNA levels seemed to be increased in EAE mice, the differences between the two groups showed no statistical significance (Figure 8A). We also collected PBMCs of SLE and RA patients for further validation. Compared with HCs, the expression levels of *BST2* and *IFIH1* were both higher in SLE and RA, while *MX1* was only higher in RA. The *GBP1, TRIM22,* and *CXCL10* have no significant change in mRNA levels in SLE or RA patients when compared with those in the HC group (Figure 8B).

**FIGURE 9:**
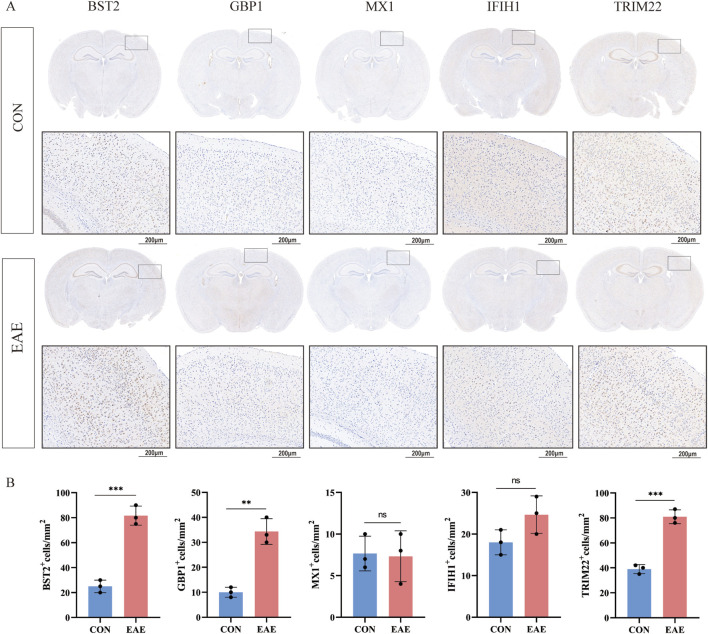
The expression of five shared genes in EAE mice brain. **(A)** Representative IHC staining images of *BST2, GBP1, MX1, IFIH1, and TRIM22* in control and EAE mice. **(B)** Comparison of protein expression differences of five hub genes according to IHC results. **p < 0.01, ***p < 0.001.

As shown in Figure 8C, there was a moderate positive area of *BST2* in glial cells, neuropil and neuronal cells in healthy human cortex. *GBP1* was lowly expressed in endothelial cells, while not detected in glia and neuronal cells. *MX1* expression was enriched in glial cells. *IFIH1* was not detected in glial cells, and it was expressed at a moderate level in neuronal cells. *TRIM22* were hard to detect in the glial cells. The IHC results for *CXCL10* are not shown in the HPA database. Based on mRNA expression data and findings from the HPA database, we conducted IHC analysis on brain sections from three control and three EAE mice to further ascertain the differential protein-level expression of five pivotal shared genes, with the exception of *CXCL10*. Likewise, the protein expression level of *BST2* (*p* < 0.001), *GBP1* (*p* < 0.01), and *TRIM22* (*p* < 0.001) were remarkably higher in EAE mice (Figures 9A, B).

## Discussion

MS is currently believed to be a neuroinflammatory disorder mediated by immune dysregulation, with both central and peripheral immune components contributing to disease development. However, its specific pathogenesis remains unclear. Patients often present with or develop multiple systemic conditions, including other AIDs, prior to or concurrent with symptom onset, which suggests a potential common pathogenic mechanism in these AIDs. Research indicates that comorbid conditions can lead to delays in MS diagnosis, affecting treatment options, quality of life, and even disability and mortality rates (Nociti and Romozzi, 2022). Autoimmune comorbidities might share a biological basis with MS, such as immune dysregulation and genetic susceptibility, possibly unveiling new therapeutic targets. An increasing body of research supports the interrelationship between various AIDs. In the present study, combining with multiple transcriptional analyses, we found that *BST2, GBP1, MX1, IFIH1, TRIM22, and CXCL10* as key diagnostic biomarkers. And further validation with animal models and clinical samples also supported these findings. Our results provide potential shared biomarkers for MS, SLE, and RA, and shed light on further exploration in these molecules involved in the development in these diseases.

WGCNA analysis revealed several gene modules associated with MS, SLE, and RA. The intersection of module genes resulted in 76 common risk genes of interest. Functional enrichment analysis indicated that these genes were mainly concentrated in “Epstein-Barr virus infection”, “TNF signaling pathway”, and “Cytokine-cytokine receptor interaction” in KEGG pathways. It is suggested that this is related to the immune inflammatory response. A previous study reported that people with mononucleosis, which is caused by the EB virus, are at higher risk for MS (Lanz et al., 2022). Lupus erythematosus and RA, like MS, have been significantly associated with EBV infection in epidemiological studies. Inflammatory factors, the TNF signaling pathway, and immune dysfunction are also involved in the development of the three diseases (Picon et al., 2021). In addition, cytokine-cytokine receptor interactions are involved in innate and acquired inflammatory host defense, angiogenesis, *etc.* GO terms significantly associated with BP are numerous and include “response to virus”, “defense response to virus”, and “regulation of leukocyte differentiation”, which are involved in the pathogenesis of MS, SLE, and RA. In addition, MF analysis and Reactome enrichment analysis of DEGs were closely related to antiviral and inflammatory responses. Taken together, these findings suggest that the significant role of these genes in immune regulation and inflammatory responses, which may explain the similar clinical manifestations observed in these diseases.

PPI network analysis identified 11 hub genes with potential roles in the diseases. The SVM-RFE algorithm in machine learning can eliminate redundant factors, retaining only variables relevant to the outcomes, showing broad potential applications in feature ranking and selection of meaningful features for classification purposes. Among the most effective feature selection methods, SVM-RFE has successfully been used to identify hub genes in various diseases (Zheng et al., 2022). In our study, we identified 6 hub genes (*BST2, GBP1, MX1, IFIH1, TRIM22, CXCL10*) by employing three machine learning methods (SVM-RFE, Lasso, and Random Forest). The candidate biomarkers selected by machine learning methods have significant clinical potential and may aid in the early diagnosis and personalized treatment of the diseases. We constructed a nodal graph and evaluated its predictive value for MS patients with concurrent SLE and RA Subsequent external validation with two additional datasets indicated a close association of these six hub genes with MS progression and demonstrated good predictive value for MS in SLE and RA patients, suggesting their potential role in the pathogenesis of MS.

SLE and RA are AIDs with a wide range of disease manifestations. Both innate and acquired immune mechanisms have been reported to be accelerated or suppressed, as described in the literature. Our results of CiberSort analysis were also in line with most previous studies in MS (Schafflick et al., 2020). We further investigated the relationship between the identified genes (*BST2, GBP1, MX1, IFIH1, TRIM22, CXCL10*) with the immune system. *BST2*, also known as *CD317*, is a type II integral membrane protein primarily involved in interferon-mediated antiviral pathways. Studies have reported that this protein anchors enveloped viruses to the surface of infected cells via its N-terminal transmembrane domain and C-terminal GPI anchor, inhibiting viral budding and thereby suppressing viral release. *BST2* serves as a host intrinsic antiviral factor, offering a new target for antiviral drug development. Apart from its role in inhibiting viral release, *BST2* also functions as an innate immune sensor during viral infections, activating NF-κB through interaction with ILT7/LILRA4 to induce inflammatory responses (Swiecki et al., 2013). Enrichment analysis of proteomic data revealed mapping of type I interferon signaling and neutrophil activation networks to both male and female SLE, with higher levels of neutrophil activation observed in male SLE compared to female SLE. Western blot confirmed the higher abundance of *PGAM1, BST2, and SERPINB10* involved in neutrophil activation in male SLE compared to female SLE (Cai et al., 2022). We observed significantly reduced methylation of interferon-regulated genes, including *IFIT1, IFIT3, MX1, STAT1, IFI44L, USP18, TRIM22, and BST2*, in naïve CD4^+^ T cells of lupus patients, indicating epigenetic transcriptional accessibility at these gene loci (Coit et al., 2013).

Viral infection triggers the expression of IFNs and interferon-stimulated genes (ISGs), which are crucial for regulating antiviral responses. *GBP1* is an ISG that exhibits antiviral activity against various viruses (Bender et al., 2024). In infected tissues, infected cells activate their own inflammatory response and release innate and adaptive immune stimulatory cytokines, such as interferon IFN-γ, to halt pathogen dissemination. Surrounding uninfected cells, in order to prevent their own infection, preemptively initiate a cascade of ISGs to establish robust antipathogenic activity. Although this process can potentially lead to self-damage, uninfected cells typically do not undergo excessive inflammatory reactions. The significant expression of *GBP1* maintains cellular viability under IFN-γ treatment (Fisch et al., 2023). qRT-PCR analysis of *GBP1, CXCL10, and MX1* mRNA expression in peripheral blood mononuclear cells of SLE patients revealed significantly higher levels in SLE patients compared to healthy donors (Liu et al., 2018). Inflammatory RA synovium, CXCL10^+^ CCL2^+^ inflammatory macrophages exhibit distinct profiles of proinflammatory and interferon response genes, including elevated levels of CXCL10, CXCL9, CCL2, CCL3, GBP1, STAT1, and IL1B (Zhang et al., 2021).

*MX1* gene belongs to a class of ISGs. *MX1* is an important antiviral protein that can inhibit the replication of various RNA viruses and some DNA viruses. The expression of the *MX1* gene is tightly regulated by type I and type III interferons. Upon binding of interferons to their receptors, downstream JAK1 and TYK2 undergo cross-phosphorylation and activation. Subsequently, activated JAKs phosphorylate downstream signaling molecules STAT1 and STAT2, inducing the formation of a STAT1-STAT2 heterodimer. This heterodimer then recruits interferon regulatory factor 9, forming the trimeric complex ISGF3, which upon nuclear translocation binds to interferon response elements, promoting MX1 expression (Haller and Kochs, 2020). In patients with MS undergoing IFN-β therapy, there is a significant increase in the expression of two key IFN-regulated genes, IFI44 and MX1, compared to newly diagnosed MS patients. Furthermore, the expression of IFI44 and MX1 after IFN-β treatment may be positively correlated, serving as responsive indicators of IFN-β therapy. The IFI44/MX1 axis may be one of the crucial regulatory factors in disease following IFN-β therapy (Jabbari et al., 2023). In kidney tissue of MRL/lpr mice and peripheral blood of LN patients, KLF5 and MX1 are both highly expressed (Tao et al., 2023).

*IFIH1* gene, which encodes the cytosolic viral RNA receptor that activates type I interferon signaling, is considered a risk factor for various AIDs, including classical psoriasis. A recent study suggests that gain-of-function mutations in the *IFIH1* gene encoding *MDA5* lead to upregulated type I interferon responses, and individuals harboring these mutations exhibit phenotypes consistent with AIDs. It is speculated that AID may be triggered by viral infections in genetically susceptible individuals. Recent research has also shown that mutations in the *MDA5* helicase domain can result in spontaneous SLE in mice. These *IFIH1 (MDA5)* mutation alleles can activate Mitochondrial Antiviral Signaling Protein-dependent signaling pathways in the absence of viral infection or without bound viral RNA ligands (Wu et al., 2021). The *IFIH1* rs1990760 T allele is associated with susceptibility to T1D, SLE, MS, and RA. Our results further demonstrate that common genetic factors underlie multiple AIDs (Cen et al., 2013).

The *TRIM* family members are characterized by an N-terminus containing three conserved domains, including a zinc finger domain (RING finger), one or two B-box domains, a coiled-coil domain, and a variable C-terminus, hence also known as the RBCC (RING, B-box, and Coil-coil) family. The RING domains of most TRIM proteins possess E3 ubiquitin ligase activity, playing crucial roles in regulating viral replication, host antiviral innate immune responses, and inflammatory reactions (Koepke et al., 2021). Several TRIM molecules are known to inhibit the process of IAV infection. TRIM19, TRIM22, and TRIM56 exhibit broad-spectrum antiviral activity, with TRIM22 directly targeting the influenza virus NP protein, mediating NP protein ubiquitination and its degradation via the proteasomal pathway (van Gent et al., 2018). The TRIM protein family, also known as the Tripartite Motif protein family, previous studies on the expression patterns of human and murine TRIM families have found that certain genes in this family, such as TRIM5, TRIM19, TRIM22, and TRIM25, are induced and upregulated by IFNs. Following viral invasion, the activated IFN response pathway induces the expression of TRIM proteins. TRIM-α can directly bind to viral capsid proteins upon cell entry, inhibiting viral RNA uncoating. The expression of TRIM is a response to IFN stimulation and is essential for controlling viral infections. Changes in TRIM22 expression are also associated with diseases such as MS, cancer, and other AIDs (Kelly et al., 2014). These findings confirm the significant role of these six immune-related genes in the development of MS. Importantly, these six genes are closely associated with dysfunction of different types of immune cells in immune infiltration, suggesting their potential utility in predicting the risk of MS coexisting with SLE and RA, and in reducing immune responses during adjunctive therapy.

MS is considered the cornerstone of central nervous system autoimmune demyelinating diseases, while systemic AIDs serve as important analogs of MS. Our research indicates that potential systemic autoimmunity is not uncommon in patients undergoing possible CNS demyelination assessment (Karathanasis et al., 2022). Here, we assessed the pathogenic genes shared by MS, SLE, and RA: *BST2, GBP1, MX1, IFIH1, TRIM22, and CXCL10*. Our study findings suggest that IFN-related gene expression and pathways are common features in the pathogenesis of MS, SLE, and RA. The activation of the type I interferon pathway may be a key mediator of systemic AIDs presenting with features similar to MS, providing potential avenues for future drug development to enhance the diagnosis and treatment of comorbidities of MS with rheumatic conditions. However, our study has several limitations. Firstly, the diversity of sample types in the selected dataset may introduce bias, and further validation is needed with more clinical samples. Sencondly, although the validation dataset GSE123496 was used to analyze predictive value, critical biomarkers and underlying mechanisms still require validation in experimental studies. Finally, the shared pathogenic mechanisms of MS with other AIDs, such as autoimmune thyroid diseases and psoriasis, still require further exploration.

## Conclusion

This study identified key shared genes among MS, SLE, and RA, proposing them as potential early diagnostic biomarkers. Our findings provide the foothold for future studies on potential crucial candidate genes for MS in SLE or RA patients. Additionally, the dysregulated immune cell proportions and immune checkpoint expressions in MS highlight the potential of these genes in disease pathogenesis and treatment.

## Data Availability

The original contributions presented in the study are included in the article/Supplementary Material, further inquiries can be directed to the corresponding authors.

## Abbreviations

MS, Multiple sclerosis; CNS, Central nervous system; AIDs, Autoimmune diseases; SLE, Systemic lupus erythematosus; RA, Rheumatoid arthritis; ILCs, Innate lymphoid cells; GEO, Gene Expression Omnibus; WGCNA, Weighted gene coexpression network analysis; MAD, Median absolute deviation; TOM, Topological overlap matrix; scRNA-seq analysis, Single-cell RNA-sequencing analysis; FDR, False discovery rate; GO, Gene Ontology; BP, Biological process; CC, Cellular component; MF, Molecular function; KEGG, Kyoto Encyclopedia of Genes and Genomes; PPI, Protein-protein interaction; LASSO regression, Least absolute shrinkage and selection operator regression; SVM-RFE, Support vector machine-recursive feature elimination; qRT- PCR, Quantitative real-time polymerase chain reaction; ISGs, Interferon-stimulated genes; MOG35-55, Myelin Oligodendrocyte Glycoprotein 35–55.
