# Supplementary material for: Exploring the shared gene signatures and mechanism among three autoimmune diseases by bulk RNA sequencing integrated with single-cell RNA sequencing analysis
Source: Front Mol Biosci. 2025 Jan 7;11:1520050. doi: 10.3389/fmolb.2024.1520050 (PMC11746102; doi:10.3389/fmolb.2024.1520050)
Supplement: Supplementary file 1 [file DataSheet1.docx]

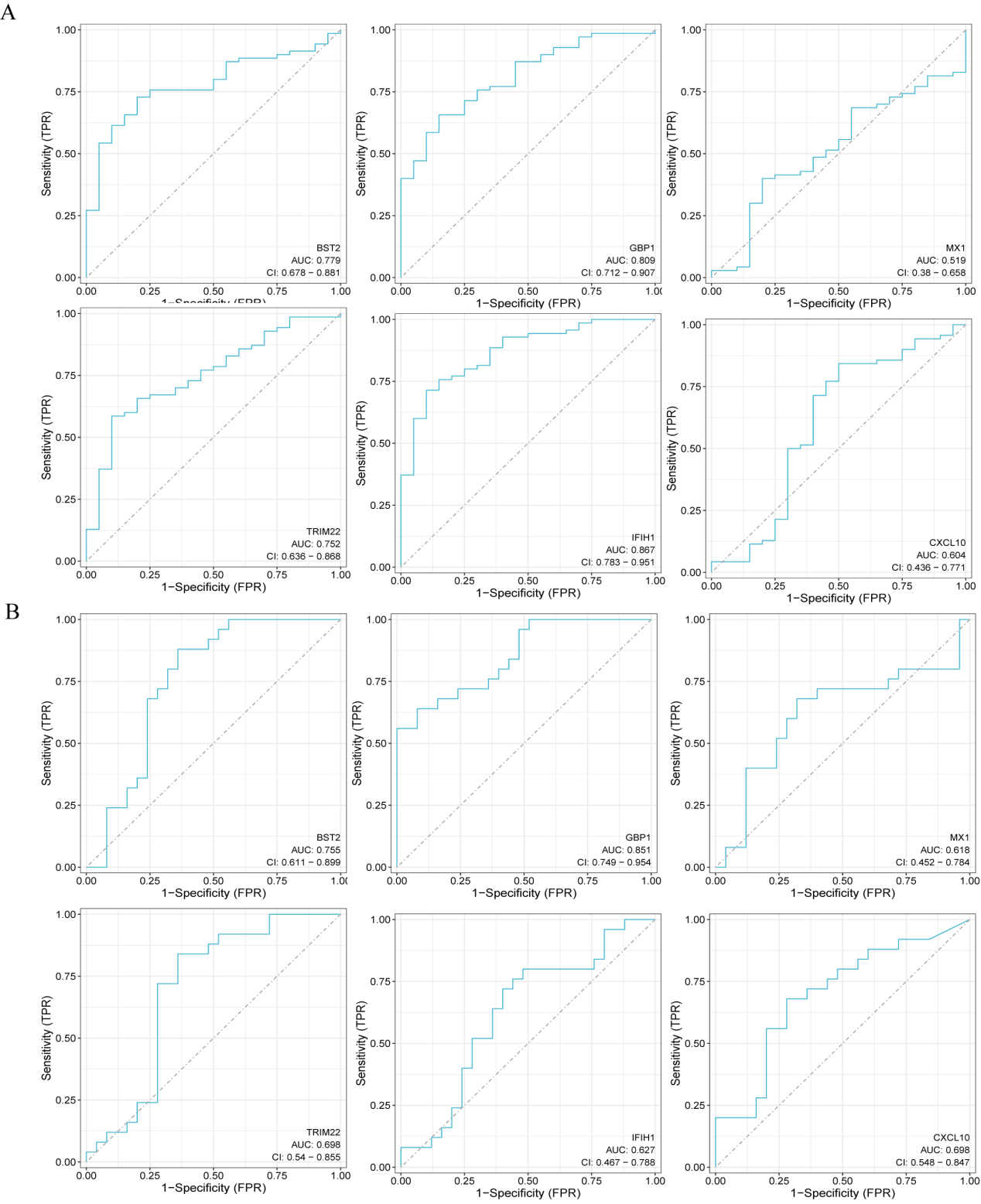


**Fig. S1.** Diagnostic values of candidate biomarkers of MS progression in SLE and RA assessed by ROC curve.

(A) The ROC curves of the 6 candidate biomarkers in the intergrated two datasets of GSE135511

and GSE108000. The AUCs and 95% CIs are displayed at the bottom of each panel. (B) The ROC curves of the 6 candidate biomarkers in the GSE123496 validation dataset.

Abbreviations: MS,multiple sclerosis;SLE, systemic lupus erythematosus;RA, rheumatoid arthritis; BST2, bone marrow stromal cell antigen 2; GBP1, Guanylate Binding Protein 1; MX1, MX dynamin like GTPase 1; IFIH1, Interferon Induced With Helicase C Domain 1; TRIM22, Tripartite Motif Containing 22; CXCL10, C-X-C Motif Chemokine Ligand 10; ROC, receiver operating characteristics curve; AUC, area under curve; CI, confidence interval.

**TABLE S1.** Primer sequences

| GENE | Sequence |
| --- | --- |
| mGAPDH-F | TGACCTCAACTACATGGTCTACA |
| mGAPDH-R | CTTCCCATTCTCGGCCTTG |
| mBST2-R | TGTCGCAATGTCACCCATCT |
| mBST2-R | CTTCTCAGTCGCTCCACCTC |
| mGBP1-F | TGGAACGTGTGAAAGCTGAG |
| mGBP1-R | TGACAGGAAGGCTCTGGTC |
| mCXCL10-F | CGTTGAGATGCACGTATTGCC |
| mCXCL10-R | AGCATCACTCCAGTTAGCCC |
| mTRIM22-F | CAAGATCGCCTGGCGACAG |
| mTRIM22-R | CAGCTGTGTAGCCCAGGTTA |
| mIFIH1-F | CCCGAAAGACACACAGAATCAGA |
| mIFIH1-R | AGGCTTCCCATTGCCTGCAT |
| mMX1-F | ATACGACAGCCGGTTGTTCA |
| mMx1-R | CCTGAGCGGCCACGATATTT |
| GAPDH-F | GGTGAAGGTCGGAGTCAACG |
| GAPDH-R | CAAAGTTGTCATGGATGATCC |
| GBP1-F | AAGGGCATCTGGATGTGGTG |
| GBP1-R | TCTGGTTGTCACCCTTCTGC |
| BST2-F | CCTGTAGAGACGGGTTGCG |
| BST2-R | CTGAAGGGTCACCACGGTC |
| CXCL10-F | CCACGTGTTGAGATCATTGCC |
| CXCL10-R | TCACTCCAGTTAAGGAGCCC |
| TRIM22-F | CCGCCTGGAAGATCGAGAG |
| TRIM22-R | CTGTAGCTGCTGCCAGGTTA |
| IFIH1-F | CCCAGAAGACAACACAGAATCAGA |
| IFIH1-R | ACTTCCCATGGTGCCTGAAT |
| MX1-F | AGACAGCCGGTTGTTTACCA |
| Mx1-R | CCCGGCCACGATACTGATTT |

m:mouse
